# Persistently Increased Systemic ACE2 Activity Is Associated With an Increased Inflammatory Response in Smokers With COVID-19

**DOI:** 10.3389/fphys.2021.653045

**Published:** 2021-05-28

**Authors:** Gagandeep Kaur, Shaiesh Yogeswaran, Thivanka Muthumalage, Irfan Rahman

**Affiliations:** Department of Environmental Medicine, University of Rochester Medical Center, Rochester, NY, United States

**Keywords:** COVID-19, inflammation, ACE2, furin, smokers

## Abstract

**Background:** Tobacco smoking is known to be involved in the pathogenesis of several cardiopulmonary diseases. Additionally, smokers are highly susceptible to infectious agents due to weakened immunity. However, the progression of lung injury based on SARS-CoV-2-mediated COVID-19 pathogenesis amongst smokers and those with pre-existing pulmonary diseases is not known. We determined the systemic levels and activity of COVID-19 associated proteins, cytokine/chemokines, and lipid mediators (lipidomics) amongst COVID-19 patients with and without a history of smoking to understand the underlying susceptible factor in the pathogenesis of COVID-19.

**Methods:** We obtained serum from healthy (CoV−), COVID-19 positive (CoV+), and COVID-19 recovered (CoV Rec) subjects with and without a history of smoking. We conducted a Luminex multiplex assay (cytokine levels), LC/MS (eicosanoids or oxylipin panel), and ACE2 enzymatic activity assays on the serum samples to determine the systemic changes in COVID-19 patients.

**Results:** On comparing the levels of serum ACE2 amongst COVID-19 (positive and recovered) patients and healthy controls, we found a pronounced increase in serum ACE2 levels in patients with COVID-19 infection. Furthermore, ACE2 enzyme activity was significantly increased amongst COVID-19 patients with a smoking history. Also, we analyzed the levels of Angiotensin 1–7 (Ang1–7) peptide, the product of enzymatic action of ACE2, in the serum samples. We found significantly high levels of Ang1–7 in the serum of both CoV+ and CoV Rec patients. Our data further demonstrated a smoking-induced increase in serum furin and inflammatory cytokine [IFN_γ_(*p* = 0.0836), Eotaxin (*p* < 0.05), MCP-1 (*p* < 0.05), and IL-9 (*p* = 0.0991)] levels in COVID-19 patients as compared to non-smoking controls. Overall, our results show that smoking adversely affects the levels of systemic inflammatory markers and COVID-19 associated proteins, thus suggesting that COVID-19 infection may have severe outcomes amongst smokers.

## Introduction

The current pandemic of Coronavirus disease 2019 (COVID-19) has emerged as a significant public health threat worldwide. Viral pneumonia and acute respiratory failure are the most common clinical manifestations of severe COVID-19, featuring fever, cough, hypoxemia, dyspnea, and bilateral infiltrates on chest radiography (Brosnahan et al., 2020). In humans, SARS-CoV2 binds to the ACE2 (angiotensin-converting enzyme 2) receptor. ACE2 is abundant in the lung epithelium, specifically type II pneumocytes, goblet cells, nasal epithelial/ciliated cells and oral mucosa (Kaur et al., 2020; Sungnak et al., 2020; Ziegler et al., 2020). It converts angiotensin II (Ang II) to angiotensin 1–7 (Ang1–7), a metabolite known to exert vasodilatory effects and oppose the actions of the ACE2 homolog, ACE, within the cell. However, since the onset of this pandemic, ACE2 is being widely studied in the context of COVID-19 (Zamorano Cuervo and Grandvaux, 2020). In this regard, the specific nature of changes in ACE2 levels and activity induced by SARS-CoV-2 infection remains elusive.

With the continual spreading of the virus and reports of novel mutant strains causing further alarm and panic, questions regarding COVID-19 risk factors have become even more urgent. While old age, heart disease, hypertension, and diabetes are the universally accepted risk factors for COVID-19, many other factors are subject to debate. One such risk factor is smoking. While initial reports on COVID-19 risk factors have indicated little to no risk amongst smokers, recent data suggest otherwise (Grundy et al., 2020; Kaur et al., 2020). A meta-analysis of 15 studies with a total of 2473 confirmed COVID-19 patients reported that COPD patients (63% vs. 33.4 in people without COPD) and current smokers (22%) are at a higher risk of severity and mortality due to COVID-19 compared to disease-free and non-smoking individuals, respectively (95% confidence interval) (Alqahtani et al., 2020; Leung and Sin, 2020). Similar findings were reported by Patanavanich and Glantz (2020), whose research suggested that smoking nearly doubles the rate of COVID-19 progression amongst patients. Despite these findings, the exact pathogenesis of COVID-19 and the clinical features resulting in severe outcomes amongst smokers is largely unexplored.

Considering this lack of research and the growing call of concern for further understanding about the COVID-19 risk-factors, we investigated whether a variation in systemic markers for inflammation and COVID-19 infection existed between smokers and non-smokers. We also studied the gender-based differences in the levels and activity of COVID-19 related proteins (ACE2 and Furin) to understand disease pathogenesis amongst both sexes. Evidence from previous literature has suggested upregulated levels of ACE-2 in the lungs of smokers (Cai et al., 2020; Leung et al., 2020). However, there is no evidence correlating this increased expression to COVID-19 disease development and associated susceptibility. Our study investigates the relationship between COVID-19 associated proteins and shows a marked increase in the lung-to-serum inflammatory spillover amongst COVID-19 positive patients with a smoking history compared to controls. Lipid profiling further shows slight increases in the levels of prostaglandins (F2_α_), 15-hydroxyeicosatetraenoic acid (15-HETEs) and 5(6)-epoxyeicosatrienoic acid (5(6)-EET) in serum collected from COVID-19 positive patients as compared to COVID-19 recovered individuals.

## Materials and Methods

### Ethics and Approvals

All the procedures performed in this study comply with the protocols approved by the Institutional Biosafety committee at the University of Rochester Medical Center, Rochester, NY, with an approval number Rahman/102054/09-167/07-186. The patient samples and information used in this study were procured from a commercial provider- BioIVT (Westbury, NY, United States). All the laboratory procedures were performed per the regulations specified by the BSL2+ level of containment for Clinical and Research Safety.

### Human Blood Serum Collection

Sera from healthy (CoV−), COVID-19 positive (CoV+), and COVID-19-Recovered (CoV Rec) subjects were obtained from BioIVT (Westbury, NY, USA). The samples were collected between April-July, 2020. Disease status was confirmed by BioIVT using RT-PCR and/or antibody (Diazyme serological assay) test for COVID-19. The patients grouped as “COVID-19 recovered” were tested “positive” and were determined convalescent 30 days post symptoms per the CDC guidance. The patient population was categorized based on smoking status; both current and previous tobacco smokers were considered “Smokers” for subsequent analyses. The patient population that has “never smoked” is termed as non-smokers. The characteristics of the study subjects used for the experiments are presented in Table 1.

**TABLE 1:** Characteristics of study subjects.

**Characteristics**	**CoV−**	**CoV+**	**CoV Rec**	***p*-value^*a*^**
N	18	16	21	
Age (years), mean (SD)	36.055 (8.39)	39.75 (14.68)	45.5 (13.81)	0.4836
Male Sex, n (%)	9 (50)	8 (50)	10 (47.62)	0.0667
Smoker, n (%)	9 (50)	3 (18.75)	6 (28.57)	0.9333
Caucasian**	9 (50)	13 (81.75)	20 (90.23)	>0.9999

^a^Kruskal-Wallis test. **p < 0.05 per Chi-square test.

### Assessment of Pro-inflammatory Mediators in Blood Sera Using Luminex Multiplex Assay

The levels of pro-inflammatory cytokines/chemokines like MCP-1, IL-8, IFN_γ_, TNF-α, and IL-7 in the sera were measured by Luminex multiplex assay using Bio-Plex Pro^TM^ Human cytokine 27-plex assay (Cat#M500KCAFOY, Bio-Rad Labs, Hercules, CA) as per the manufacturer’s directions. Blood sera were diluted fourfold. The levels of 27 pro-inflammatory mediators were measured using the Luminex FlexMap3D system (Luminex, Austin, TX) and plotted in pg/mL.

### Assessment of Furin Levels Using ELISA

To determine the level of Furin in sera collected from CoV−, CoV+ and CoV Rec subjects, we employed a Human Furin ELISA kit (Cat # ab113322, Abcam, Cambridge, MA) as per the manufacturer’s protocol. Colorimetric detection was performed at 450 nm using Cytation 5 microplate reader (BioTek Instruments, Inc. Winooski, VT). Furin levels were expressed as pg/mL.

### Assessment of ACE2 Levels Using ELISA

We employed a commercially available Human ACE2 ELISA kit (Cat#ELH-ACE2, Ray-Biotech, Peachtree Corners, GA) to determine the ACE2 levels in the patient’s serum samples, per manufacturer’s directions. Colorimetric detection was performed at 450 nm using Cytation 5 microplate reader (BioTek Instruments, Inc., Winooski, VT) and serum ACE2 levels were measured in ng/mL.

### Assessment of ACE2 Activity

We utilized the ACE2 Activity Assay kit (Cat #: K897 BioVision, Milpitas, CA, United States) to determine the ACE2 activity in the human serum samples. The assay was performed as per the manufacturer’s instructions. In brief, serum samples were diluted by adding equal volumes of ACE2 Lysis Buffer and ACE2 Assay Buffer. In addition to the appropriate standards and controls (positive, negative, and background), the diluted serum samples were then added to a 96-well plate. After that, we added ACE2 substrate to both sample and control wells. Subsequently, fluorescence was measured at an excitation maximum of 320 nm and an emission maximum of 420 nm using Cytation 5 microplate reader (BioTek Instruments, Inc., Winooski, VT). Total protein content per sample was determined using the Bradford protein assay kit (Thermo Fisher Scientific, Waltham, MA). Sample ACE2 activity for each sample was calculated using the following formula:

$$\mathrm{Sample}\,\mathrm{ACE2}\,\mathrm{Activity}={\text{B}}^{*}\,\text{D}/\left(\Delta \,{\text{T}}^{*}\,\text{P}\right)$$

where, B = Released MCA (cleaved product of ACE2 substrate) in Sample based on standard curve slope (pmol), ΔT = Reaction time (in min), P = Sample used (in mg), and D = Sample Dilution factor.

### Assessment of Angiotensin II Levels Using ELISA

To determine the levels of Angiotensin II in sera collected from COVID-19 negative, COVID-19 positive, and COVID-19 recovered subjects, we utilized the Human Angiotensin II Competitive ELISA kit (Cat #: RAB0010, Sigma-Aldrich, St. Louis, MO) as per the manufacturer’s protocol. The serum samples were diluted 20-fold for this experiment. Colorimetric detection was performed at 450 nm using Cytation 5 microplate reader (BioTek Instruments, Inc. Winooski, VT). Angiotensin II levels were expressed as pg/mL.

### Assessment of Angiotensin I-7 Levels Using ELISA

We utilized the Human Angiotensin 1–7 ELISA kit (Cat #: NBP2-69078, NOVUS Biologicals, Littleton, CO) to determine the serum Ang1–7 levels as per the manufacturer’s protocols. The serum was diluted 5-fold for this study. Colorimetric detection was performed at 450nm using the Cytation 5 microplate reader (BioTek Instruments, Inc. Winooski, VT). Angiotensin 1–7 levels were expressed as pg/mL.

### Determination of Serum Eicosanoid/Oxylipins Levels Through Lipidomic Analysis

Serum eicosanoid/oxylipin profiling was outsourced to and performed by Cayman Chemical (Ann Arbor, MI). Lipid profiling was done using ultraperformance liquid chromatography in tandem with mass spectroscopy (UPLC-MS/MS). Lipidomes were prepared using serum from six different age-, sex-, and smoking status-matched patients from each group.

Nomenclature: The abbreviations used for various classes of lipids include the following:

6-keto PGF1_α_: 6-keto Prostaglandin F_1α_, TXB2: Thomboxane B_2,_ PGF_2α_: Prostaglandin F_2α_, PGE2: Prostaglandin E_2_, 12-HHTrE: 12-Hyrdoxyheptadecatrenoic acid, LTB4: Leukotriene B_4_, LXA4: Lipoxin A_4_, HETE: Hydroxyeicosatetraenoic acid, EET: epoxyeicosatrienoic acid, HODE: Hydroxyoctadecadienoic acid and HDHA: Hydroxy Docosahexaenoic Acid.

Lipid extraction: Lipid extraction from the serum samples was performed by protein precipitation followed by solid-phase extraction (SPE). Protein precipitation was performed by the addition of H_2_O: Acetonitrile solution to each sample. After that, SPE was performed using Strata-X cartridges (33 μm, 200 mg/10 mL; Phenomenex, PA). The extracted lipids were finally dissolved in water/acetonitrile 60:40 (v:v) solution. To prepare the calibration curves, a mixture of the 20 calibration standards was prepared in methanol.

UPLC-MS/MS: Equal volumes of calibration standards and samples was added to Kinetex (2.6 μm C18 100 Å 100×2.1 mm, Phenomenex OOD-4462-AN) column, and Reverse phase liquid chromatography (LC) using Sciex ExionLC Integrated System was used for lipid separation. The lipid quantification in the samples was performed using Sciex 6,500+. The total amount of eicosanoids present in each sample was determined using MultiQuant software (Sciex). The lipid abundance ratios were calculated in terms of log base twofold change and plotted as a heat map using GraphPad Prism 8.0.

### Statistical Analyses

All statistical calculations were performed using GraphPad Prism 8.0. Data was expressed as mean ± SE. Comparisons between two data groups were made using unpaired *t*-test or Mann Whitney’s test, while One-way ANOVA was used for multiple comparisons. Differences were considered statistically significant at ^∗^*p* < 0.05, ^∗∗^*p <* 0.01, ^∗∗∗^*p <* 0.001, and ^****^p < 0.0001 when compared with respective controls.

## Results

### Serum ACE2 Levels Are Increased by COVID-19 Infection

**FIGURE 1:**
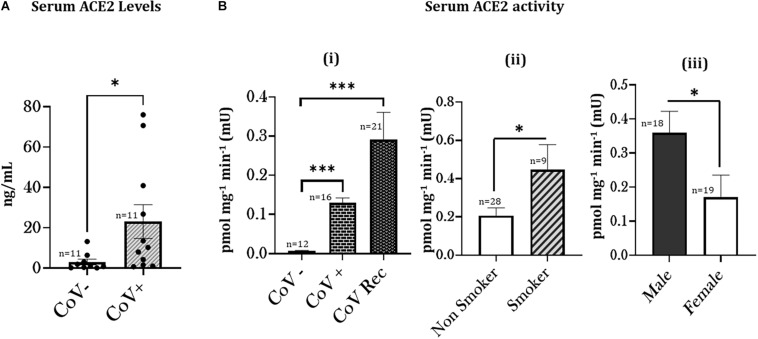
Increased ACE2 levels and activity amongst COVID-19 patients with a smoking history. **(A)** Blood serum samples from COVID-19 negative (CoV–) and COVID-19 (positive and recovered) patients were obtained and the ACE2 level was quantitatively measured. ^∗^*p* < 0.05 as per Mann Whitney’s test for pairwise comparisons. CoV+: COVID19 positive (current and recovered) patients. **(B)** Patient Sera from healthy (CoV–), COVID-19 positive (CoV+), and COVID-19 recovered (CoV Rec) subjects were obtained, and ACE2 Activity was quantitatively measured. The obtained results were plotted based on serum ACE2 activity in (i) healthy subjects vs. COVID-19 positive and COVID-19 recovered patients. Data are shown as mean ± SEM. (^∗∗∗^*p* < 0.001, as per one-way ANOVA for multiple comparisons), (ii) COVID-19 (positive and recovered) patients with or without a smoking history, and (iii) COVID-19 patients based on their gender. Data are shown as mean ± SEM. ^∗^*p* < 0.05, ^∗∗∗^ *p* < 0.001 as per unpaired *t*-test.

In humans, ACE2 binds to the SARS-CoV2 spike protein (Lan et al., 2020). In light of this, we first investigated changes in the levels of serum ACE2 amongst COVID-19 (positive and recovered) patients and healthy controls using an ELISA-based assay. Our results indicate a significant increase in the serum ACE2 levels on COVID-19 infection as shown in Figure 1A.

### ACE-2 Activity Varies as a Function of Smoking History Among COVID-19 Patients

Next, we investigated the serum ACE2 activity in the patient sera from COVID-19 positive and recovered patients and compared it to healthy subjects. We found a significant increase in the serum ACE2 activity in both COVID-19 positive and COVID-19 recovered patients. We found a higher ACE2 activity in serum from COVID-19 recovered patients compared to serum from COVID-19 positive patients (Figures 1Bi). These results show that the ACE2 activity remains persistently high amongst COVID-19 patients.

To determine the smoking-related changes in the ACE2 function amongst smokers and non-smokers, we next compared the ACE2 activities amongst COVID-19 (positive and recovered) patients with and without a smoking history. We found a significant increase in the serum ACE2 activity from COVID-19 patients with a smoking history as compared to non-smokers (Figure 1Bii). ACE2 activity was more pronounced among male patients than females (Figure 1Biii). Our results show that smoking status plays a crucial role in governing the COVID-19 related enzyme activity in human subjects, suggesting a role in individuals’ disease pathogenesis.

### Differential Levels of Angiotensin II and Angiotensin 1–7 Based on COVID-19 Status

**FIGURE 2:**
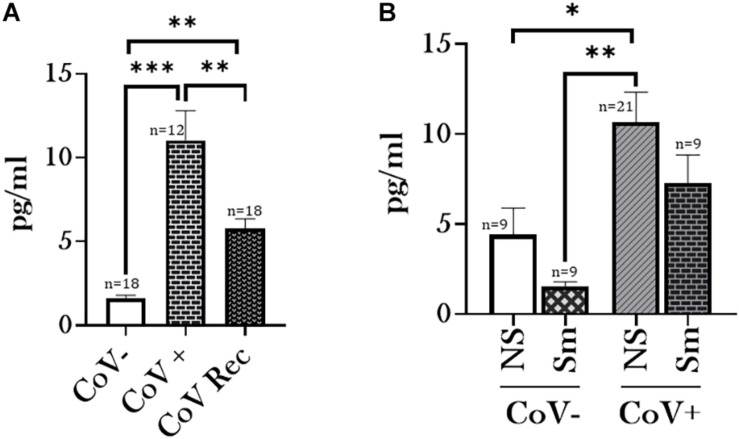
Angiotensin II levels increase amongst patients with a history of COVID-19. Blood serum samples from healthy (CoV–), COVID-19 positive (CoV+), and COVID-19 recovered (CoV Rec)subjects were obtained and Angiotensin II (AngII) levels were quantitatively measured. The obtained results were plotted based on serum levels in **(A)** CoV–, CoV+, and CoV Rec subjects and **(B)** CoV- and COVID-19 (positive and recovered) patients with and without a smoking history. Data are shown as mean ± SEM. ^∗^*p* < 0.05, ^∗∗^*p* < 0.01, and ^∗∗∗^*p* < 0.001 as per One-Way ANOVA for multiple comparisons. NS, Non-Smokers and Sm: Smokers.

**FIGURE 3:**
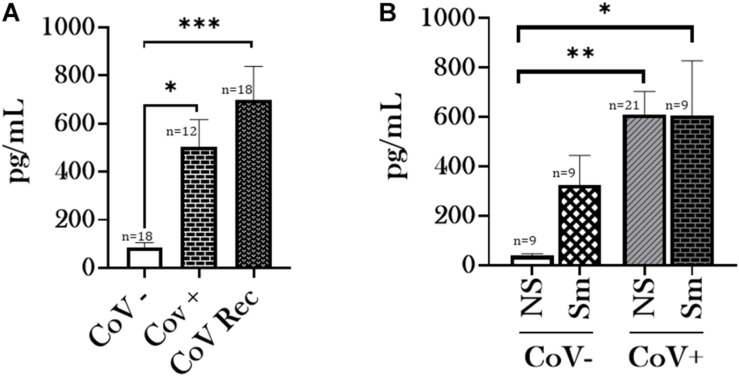
Serum Angiotensin 1–7 levels increase amongst patients with a history of COVID-19. Blood serum samples from healthy (CoV–), COVID-19 positive (CoV+), and COVID-19 recovered (CoV Rec) subjects were obtained, and Angiotensin 1–7 (Ang1–7) levels were quantitatively measured. The obtained results were plotted based on serum levels in **(A)** CoV-, CoV+, and CoV Rec subjects and **(B)** CoV- and COVID-19 (positive and recovered) patients with and without a smoking history. Data are shown as mean ± SEM. ^∗^*p* < 0.05, ^∗∗^*p* < 0.01, and ^∗∗∗^*p* < 0.001 as per One-Way ANOVA for multiple comparisons. NS, Non-Smokers and Sm: Smokers.

Having observed a differential ACE2 activity between smokers and non-smoker who have had a history of SARS-CoV-2 infection, we were next interested in comparing the Angiotensin II (Ang II) and Angiotensin 1–7 (Ang1–7) levels in our experimental groups. ACE2 catalyzes the conversion of Ang II to Ang1–7. Interestingly, our data indicated increased levels of Ang II and Ang1–7 in the serological samples from CoV+ and CoV Rec patients compared to healthy subjects (Figures 2A, 3A). It is pertinent to mention however, that increase in the Ang1–7 levels was comparatively higher (∼6-fold) (Figure 3A) than that observed for Ang II (∼4-fold) (Figure 2A) in our experiments.

We further compared the Ang II and Ang1–7 levels based on the smoking history of our study participants. Here, we observed a noticeable increase in the ACE2 substrate (Ang II) and product (Ang1–7) levels amongst non-smokers on COVID-19 infection as compared to healthy controls (Figures 2B, 3B). Such an increase was not observed between COVID-19 positive and healthy individuals with a smoking history. Of note, we did not observe any gender-based disparity in the AngII or Ang1–7 levels amongst the CoV−, CoV+, and CoV Rec subjects in our study (data not shown).

### Smoking Alters Furin Levels in Serological Samples

**FIGURE 4:**
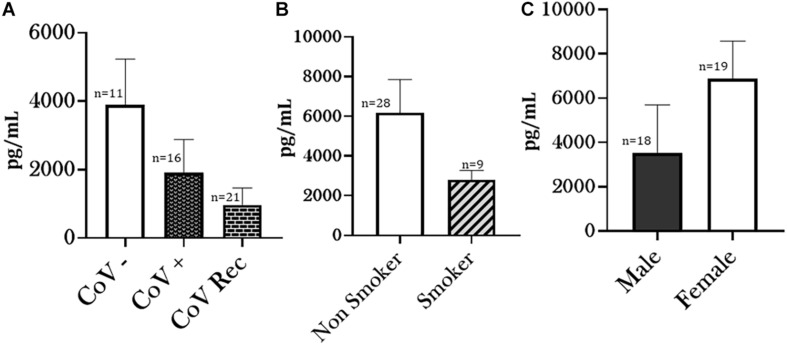
Altered Furin levels amongst COVID-19 patients. Blood serum from healthy (CoV–), COVID-19 positive (CoV+), and COVID-19 recovered (CoV Rec) subjects were obtained, and the Furin levels were quantitatively measured. The obtained results were plotted based on serum Furin levels in **(A)** healthy subjects vs. COVID-19 positive or COVID-19 recovered patients, **(B)** COVID-19 (positive and recovered) patients with or without a smoking history, and **(C)** COVID-19 (positive and recovered) patients based on their gender. Data are shown as mean ± SEM.

Another key to understanding COVID-19 virulence as a function of susceptibility to viral entry is analyzing changes in Furin levels. Unlike other Coronaviruses, SARS-CoV-2 has a lower dependence on target host-cell proteases and depends more on pro-protein convertase Furin for its viral entry (Johnson et al., 2021). Based on this, we measured Furin-levels in sera from CoV−, CoV+, and CoV Rec groups using ELISA. We observed a noticeable (though not significant) decrease in the serum Furin levels in COVID-19 recovered patient groups as compared to healthy controls. Though not significant, the serum Furin levels in CoV+ patients was also lower as compared to CoV− patients (Figure 4A). Likewise, the serum Furin level was lowered amongst smokers compared to non-smokers in patient sera collected from COVID-19 (positive and recovered) patients (Figure 4B). Though not shown here, the Furin levels were significantly higher amongst healthy smokers compared to healthy non-smokers (data not shown). Taken together, our results show that a SARS-CoV2 infection decreases the extracellular Furin levels irrespective of the smoking status.

Similarly, although not significant, Furin levels among females were elevated compared to males amongst COVID-19 (positive and recovered) patients (Figure 4C). However, due to the lack of enough gender-matched controls, we were unable to determine if such a gender-based variation is observed amongst healthy individuals.

### Infection With SARS-CoV-2 Upregulates Pro-inflammatory Cytokine Levels in Smokers

**FIGURE 5:**
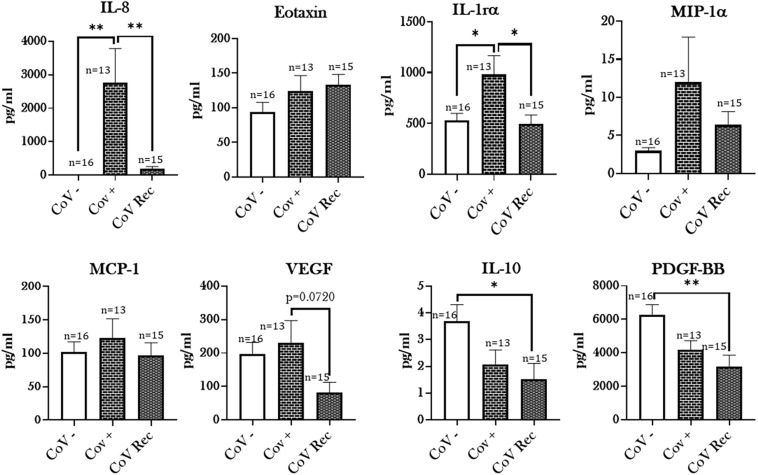
Elevated cytokine levels in COVID-19 positive patients. Blood serum samples from healthy (CoV–), COVID-19 positive (CoV+), and COVID-19 recovered (CoV Rec) subjects were obtained, and the levels of cytokines/chemokines were assessed with the help of Luminex multiplex assay. The levels of detected cytokines were plotted. Data are shown as mean ± SEM. ^∗^ *p* < 0.05, ^∗∗^ *p* < 0.01; as per One-way ANOVA for multiple comparisons.

It is well known that the severity of COVID-19 is associated with increased levels of pro-inflammatory mediators (Hojyo et al., 2020; Tang et al., 2020; Zhou et al., 2020). Given this, we analyzed the levels of 27 cytokines/chemokines in the serum samples from, CoV−, CoV+, and CoV Rec study populations using Luminex multiplex assay. Our results showed a significant increase in the levels of IL-8 and IL-1rα in COVID-19 positive patient sera compared to healthy controls (Figure 5). In contrast, the levels of IL-10 and PDGF-BB were significantly lowered amongst COVID-19 recovered patients as compared to healthy individuals. Parameters such as, IL-5, GM-CSF, IL-12(p70), and IL-15 were at non-detectable levels in the CoV+ and CoV Rec groups.

**FIGURE 6:**
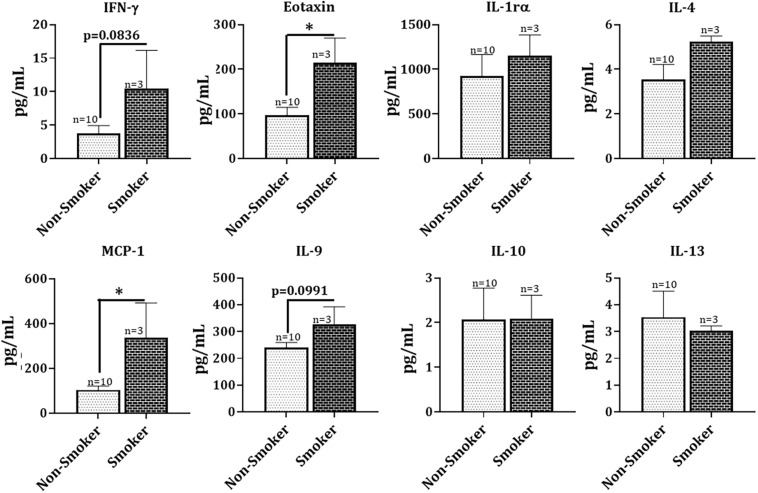
Hyperinflammation in COVID-19 positive patients with a smoking history. Levels of cytokines/chemokines as assessed with the help of Luminex multiplex assay in blood serum samples from COVID-19 positive patients with and without a smoking history were collected. Data are shown as mean ± SEM. ^∗^*p* < 0.05, as per unpaired *t*-test.

Intriguingly, when analyzing cytokine/chemokine levels in patient sera based on smoking status, a notable trend emerged. We noted a substantial increase in the production of pro-inflammatory markers like IFN_γ_ (*p* = 0.0836), MCP-1 (*p* < 0.05), and Eotaxin (*p* < 0.05) in the COVID-19 positive patient sera from those with a smoking history compared to the non-smoking controls. Furthermore, we found a moderate increase in the levels of IL-9 (*p* = 0.0991) amongst smokers infected with COVID-19 compared to COVID-19 positive non-smokers (Figure 6). It is important to mention here that the levels of inflammatory mediators in the serum did not show any gender-based variations in our study (data not shown).

### Altered Serum Lipid Profile Amongst COVID-19 Positive Patients

**FIGURE 7:**
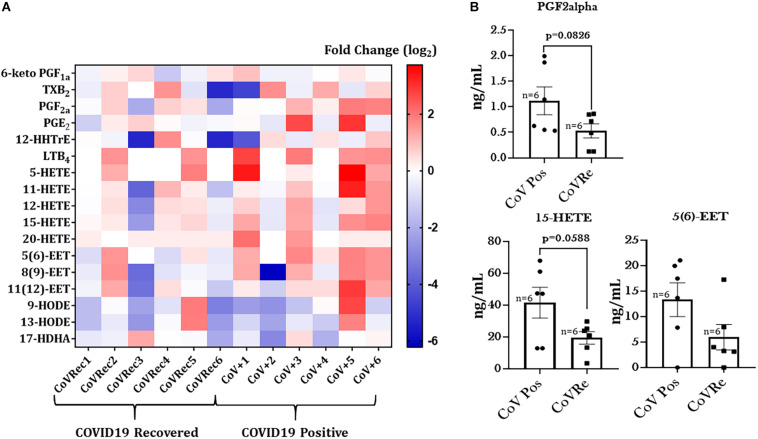
Heat map of eicosanoid levels in COVID-19 positive and COVID-19 recovered patient serum. **(A)** The fold changes in the eicosanoid levels in COVID-19 recovered patient serum as compared to COVID-19 positive serum. Each horizontal row represents the analyte in a specific lipid class, and each vertical column represents the individual sample being tested. Lipid abundance ratios are colored according to the fold changes and the color key indicates the magnitude of log2 fold change. **(B)** The actual changes in the levels (ng/mL) of oxylipins showing slight variations amongst COVID-19 positive and COVID-19 recovered patients.

It is known that infections can induce various alterations in lipid metabolism that can dampen inflammation or fight infection (Feingold and Grunfeld, 2000). Thus, we were next interested in studying the changes in the lipid profiles of patient sera from COVID-19 positive and COVID-19 recovered groups. Figure 7A depicts a generated heat map that shows alterations in the levels of the 17 most prevalent eicosanoids/oxylipins in sera from COVID-19 positive subjects compared to COVID-19 recovered individuals. Though none of the observed change was significant amongst CoV+ and CoV Rec groups, we found slight increase in the levels of PGF2_α_, 15-HETE, and 5(6)-EET in the COVID-19 positive patients as compared to the COVID-19 recovered patients (Figure 7B). The detailed account of the fold changes (Cov V+ vs. CoV Rec groups) in the levels of each of the studied lipids with *p*-values is provided in Table 2. It is pertinent to mention here that we did not find any statistically significant variations in the lipid profiles of COVID-19 positive and COVID-19 recovered patients based on age, sex, or smoking history.

**TABLE 2:** Differentially altered lipid analytes in COVID-19 positive patient sera with respective fold changes with COVID-19 recovered subjects.

**Analyte**	**Fold change**	***p*-value**
6-keto PGF1_α_	1.110515	0.656547
TXB2	1.416495	0.545928
PGF2_α_	2.109126	0.082623
PGE2	2.857159	0.193622
12-HHTrE	0.999787	0.999655
LTB4	2.770774	0.174837
5-HETE	4.558867	0.177288
11-HETE	2.911128	0.209095
12-HETE	1.631523	0.177848
15-HETE	2.139606	0.058767
20-HETE	1.586839	0.428033
5(6)-EET	2.247237	0.104477
8(9)-EET	1.969702	0.192472
11(12)-EET	2.398283	0.228698
9-HODE	1.462013	0.705169
13-HODE	1.014798	0.984878
17-HDHA	0.766199	0.537526

## Discussion

The current pandemic of COVID-19 poses a serious threat to the global public health and health care. While the efforts of rolling out an effective vaccine are ongoing, there is panic and uncertainty about the new mutant strains of SARS-CoV2 (present as six variants worldwide so far), and long-term effects of COVID-19 infections (Baric, 2020; Mercatelli and Giorgi, 2020). It is important to identify the high-risk populations and the underlying differences in the disease pathogenesis to limit the viral spread efficiently. There have been contradictory pieces of evidence regarding the susceptibility of smokers toward COVID-19 infection. Still, recent work has demonstrated the association of smoking with adverse clinical outcomes in COVID-19 and emphasized the role of serum ACE2 levels in its pathogenesis (Saheb Sharif-Askari et al., 2020; Lowe et al., 2021). In this regard, we were interested in understanding the COVID-19 disease pathogenesis amongst smokers and investigated the systemic responses against SARS-CoV2 infection in COVID-19 positive, COVID-19 recovered, and COVID-19 negative subjects.

We first investigated the ACE2 levels in the serum from COVID-19 (positive and recovered) patients and healthy subjects. We found significantly high levels of ACE2 in the serum of COVID-19 positive individuals. ACE2 is well-known as the binding receptor for the SARS-CoV2 on the host’s cell surface. Use of ACE inhibitors and ARB (Angiotensin II receptor blockers) in COVID-19 patients is being debated since the start of this pandemic (Behl et al., 2020; Kaur et al., 2020). On studying the ACE2 activity in serological samples from COVID-19 patients, we found a significant increase in the serum ACE2 activity in both COVID-19 positive and COVID-19 recovered subjects as compared to the controls. However, on comparing the ACE2 activities amongst COVID-19 patient (positive and recovered) cohorts, we found persistently increased ACE2 activity in COVID-19 recovered patients as compared to COVID-19 positive individuals. In this regard, Patel et al. (2021) have shown persistently elevated plasma ACE2 activity amongst patients with SARS-CoV2 infection. They further showed a direct correlation between disease severity and plasma ACE2 activity in their study participants. They speculate that this may be due to increased ectodomain shedding of ACE2 and could be responsible for the symptoms of prolonged ill-health amongst patients with COVID-19 (Patel et al., 2021). Our observations corroborate with these findings and point toward probable disruption of ACE2/Ang1–7/MasR, the receptor for Ang1–7 axis. Evidences from literature show that ACE2 has a protective role in the pathogenesis of ARDS (Li et al., 2016; Liu et al., 2020). In contrast, previous evidences have correlated increased serum ACE2 activity with increased cardiovascular risk and obstructive coronary artery disease (Úri et al., 2016; Ramchand et al., 2018; Ramchand and Burrell, 2020). In light of these evidences, further investigation in this area is imperative.

We also found a significantly higher ACE2 activity for COVID-19 (current and recovered) patients with a smoking history. As noted previously, increased plasma ACE2 activity has been shown amongst severe patients with a SARS-CoV2 infection (Patel et al., 2021). It could be speculated that smokers might have severe outcomes due to COVID-19. Despite this speculation, a future study with a larger sample size and age- and sex-matched controls is important to establish such a correlation. As reported previously (Sama et al., 2020); our data also indicate gender-based upregulations in ACE2 activity amongst males. These findings could correlate to the increased morbidity and mortality amongst male patients.

Since we show increased ACE2 activity in COVID-19 positive patients, we further analyzed the levels of Ang II (ACE2 substrate) and Ang1–7 (ACE2 product), in the serological samples from patients and normal subjects. Angiotensins are key physiological peptides involved in regulating several biological processes predominantly involved in the regulation of vascular tone and aldosterone secretion. Ang II levels play a pivotal role in adverse myocardial remodeling, while increased levels of Ang1–7 indicate counter-balancing the vaso-constrictive effects of Ang II (Patel et al., 2016). Our results demonstrated elevated levels of Ang II and Ang1–7 in serological samples from COVID-19 positive patients compared to healthy controls. However, we observed a ∼6-fold increase in the Ang1–7 levels in COVID-19 positive patient sera instead of a ∼4-fold increase for Ang II levels. This indicates the counter-regulatory effects of Ang1–7 in COVID-19 positive individuals, however, further investigation is required to confirm this.

In contrast, increased levels of Ang II might indicate non-ACE2 mediated production of Ang II with the help of protease, chymase, Cathepsin G, or CAGE. It is known that increased serum Ang II levels lead to increased vasoconstrictions, inflammation, fibrosis, and eventually heart failure, a common complication amongst patients with COVID-19 (Long et al., 2020; Samidurai and Das, 2020), even after the infection is long over (i.e., long Covid-19). Elevated serum Ang II levels play a crucial role in the progression of hypertension and heart failure. The levels of Ang II have been found to be elevated in optimally treated (using ACE blockers or ARB) patients of heart failure, thus adding to the adverse health effects (Jorde et al., 2000; Petrie et al., 2001; Li et al., 2004; Patel et al., 2016). In addition, Ang II action leads to activation of ADAM-17, resulting in increased ACE2 shedding (Patel et al., 2014). Ectodomain shedding refers to the release of a cell-membrane receptor from the cell surface to the extracellular space, notably in plasma/serum. Recent studies have shown that SARS-CoV2 induces ACE2 ectodomain shedding through the increased activation of disintegrin and metalloprotease, ADAM-17 (Palau et al., 2020). Although, the biological and clinical significance of this shedding has not been determined; elevation in plasma ACE2 activity in heart failure has been linked to worsened prognosis (Epelman et al., 2008; Putko et al., 2014).

The binding of SARS-CoV2 to ACE2 is preceded by cleavage by Furin (Örd et al., 2020). Furin is abundant in the respiratory tract both intracellularly and in circulation as a free enzyme. It enhances the viral ACE2-affinity by exposing the viral binding site on the S1 domain and revealing the effusion site on the S2 domain, which makes it a crucial factor in SARS-CoV2 infection (Xia et al., 2020). Evidence suggests that furin cleavage plays a potent role in the virulence of dengue, HIV and avian flu (Fitzgerald, 2020; Walls et al., 2020). Our investigations found a marked, though non-significant, reduction in the serum Furin levels on COVID-19 infection in both COVID-19 positive and recovered patients as compared to the healthy controls. However, the serum Furin levels in COVID-19 positive patients was slightly higher than COVID-19 recovered patients. To date, there have been limited studies pertaining to circulating Furin levels. Still, there have been associations established between dysregulated serum furin levels and the occurrence of metabolic diseases like diabetes, hypertension, obesity and cardiovascular disease. While lower serum furin levels were indicative of abdominal obesity and hypertension, higher furin levels were associated with diabetes and myocardial infarction (Fernandez et al., 2018; He et al., 2019, 2020; Wang Y. K. et al., 2020). We speculate that lowered furin levels indicate the increased internalization of Furin for viral cleavage; however, further work is required to ascertain the role of intracellular and extracellular Furin in the development of COVID-19.

Additionally, we found that Furin levels were lowered amongst smokers with COVID-19, though again this change was not significant. It is pertinent to mention here that in healthy smokers Furin levels were significantly increased (data not shown) compared to non-smoking controls. Our results point toward decrease in the Furin levels in COVID-19 irrespective of the smoking status.

Coinciding with the existing literature (Hirano and Murakami, 2020; Huang et al., 2020; Mahmudpour et al., 2020), we also found increased levels of cytokines/chemokines in the patient serum from COVID-19 positive subjects. Additionally, we for the first time, show significant changes in the levels of these pro-inflammatory mediators in COVID-19 positive patients with a smoking history as compared to the non-smoking controls. Elevated levels of IFN-_γ_ (*p* = 0.0836), MCP-1, and Eotaxin point toward increased inflammatory response amongst smokers. It may be possible that anti-inflammatory and pro-resolving mediators are decreased by smoking in COVID-19 patients. Of note, we also found some changes in the lipid profiles of COVID-19 positive and COVID-19 recovered patients. Viral infections cause changes in lipid metabolism and play a crucial role in regulating innate and adaptive immune responses (Heaton and Randall, 2011; Ketter and Randall, 2019). We did not find a significant change in the serum lipid profiles of COVID-19 positive and COVID-19 recovered samples, though slight variations in some of the lipids were noticed. Amongst the lipids that showed a slight increase in COVID-19 positive patients compared to COVID-19 recovered individuals were PGF_2α_, 15-HETE, and 5(6) EET. Of these, PGF_2α_ and15-HETE are bronchoconstrictors and cause lung injury (Fish et al., 1984; Martin et al., 1989; Zhu et al., 2003), whereas 5(6) EET has an anti-inflammatory role within the cells. It is difficult to speculate what could be the possible source or role of the observed changes in the serum lipid levels. However, in general, esterified eicosanoids are released by many cell types including immune cells (neutrophils, eosinophils, and macrophages), endothelial and epithelial cells (Hammond and O’Donnell, 2012; Mazaleuskaya et al., 2018).

**FIGURE 8:**
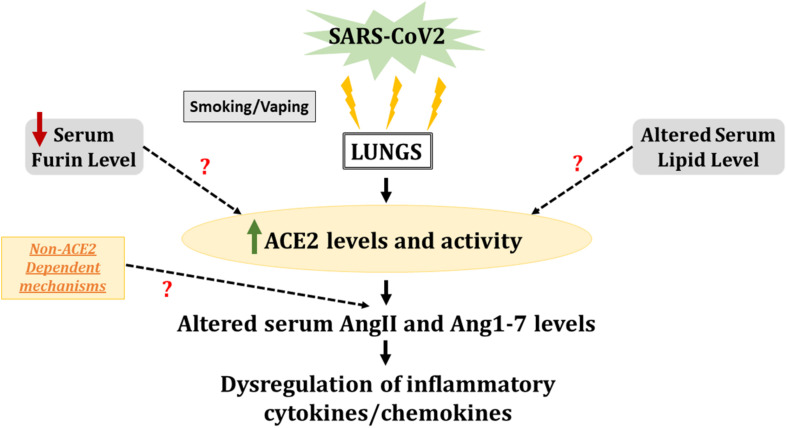
SARS-CoV2 infection leads to increased ACE2 activity and serum cytokine levels in smokers. Serum samples from healthy individuals and COVID-19 positive patients with smoking history were compared for ACE2 activity and levels of inflammatory cytokines/chemokines. The results pointed toward increased ACE2 activity and altered AngII and Ang1–7 levels in the serum of COVID-19 patients as compared to normal individuals. The altered AngII and Ang1–7 levels could also be a result of non-ACE2 dependent mechanisms which is not studied here. Increased levels of pro-inflammatory cytokines/chemokines in COVID-19 positive patients with smoking history, indicates a heightened immune response on SARS-CoV2 infection in smokers. We also found evidence for lowered serum furin and altered lipid profiles amongst COVID-19 patients, which may or may not correlate with the ACE2 activity. These alterations can lead to heightened inflammatory response and lung remodeling with smoking/vaping history.

Overall, our results provide evidence of systemic inflammatory spillover in COVID-19 positive patients (Figure 8), which is shown to be aggravated in patients who smoke. It is pertinent to mention here that pulmonary conditions like COPD and smoking-induced lung injury cause such spillovers into the systemic circulation (Tkacova, 2010; Liu et al., 2014). It will be interesting to study these inflammatory and lipid mediators along with anti-inflammatory mediators to understand the pathogenesis of SARS-CoV2 infection. However, we have not discussed this here, but the vaping population might be another group that may suffer from severe outcomes in the event of a SARS-CoV2 infection. Previous work by our group has shown gender-based variation in the ACE2 protein expression in lung tissues from C57Bl/6 mice exposed to e-cigarette (e-cig) aerosols (Wang Q. et al., 2020). E-cig use has been associated with loss of lipid homeostasis, eventually leading to pulmonary toxicity and lung injury (Madison et al., 2019; Chand et al., 2020).

Though we were able to show variations in systemic inflammatory and lipid mediators on SARS-CoV2 infection, our study had some limitations. The sample cohort used for this study was relatively small. Since we obtained our samples from a commercial source, we did not have information about disease severity, the duration of hospitalization, and medications administered to COVID-19 positive and COVID-19 recovered individuals. Similarly, there is no data about the smoking habits and pack years for current smokers or years since quitting for the ex-smokers. We intend to include a larger and more heterogeneous cohort of patients in the future. Also, due to the limited sample number we had to pool the ex- and current smokers for this study which might have introduced some confounders. The persistently elevated ACE2 activity and elevated levels of AngII and Ang1–7 raise intrigue and warrant further investigation to understand better the role of ACE2 in COVID-19 development, progress, and remission. This will have ramifications on heightened inflammatory response and lung remodeling even in recovered patients (long Covid-19) with a smoking/vaping history. Such studies are crucial in understanding the mechanistic role of intact and circulating ACE2 in COVID-19 and deduce if recombinant ACE2 could develop as a therapy.

## Conclusion

In conclusion, our data show that the systemic ACE2 activity and cytokine release are upregulated amongst COVID-19 patients with a smoking history. We also provide evidence for inflammatory systemic spillover due to SARS-CoV-2-induced COVID-19 infection, which could be crucial in identifying biomarkers in susceptible population and/or developing future therapies.

## Data Availability Statement

The original contributions presented in the study are included in the article/Supplementary Material, further inquiries can be directed to the corresponding author/s.

## Ethics Statement

The studies involving human biospecimens were reviewed and approved by University of Rochester Institutional Biosafety Committee (IBC).

## Author Contributions

GK and SY designed and conducted the experiments. GK, SY, and IR wrote, edited, and revised the manuscript. TM analyzed the lipidomic data and edited the manuscript. IR conceptually designed the overall experiments and manuscript. All authors contributed to the article and approved the submitted version.

## Conflict of Interest

The authors declare that the research was conducted in the absence of any commercial or financial relationships that could be construed as a potential conflict of interest.

## Abbreviations

COVID-19: Coronavirus Disease-2019
ACE2: Angiotensin converting Enzyme 2
GM-CSF: Granulocyte-macrophage Colony stimulating Factor
HIV: Human Immunodeficiency Virus
COPD: Chronic obstructive pulmonary disease
ARDS: Acute Respiratory Distress Syndrome.
