# Supplementary material for: Persistently Increased Systemic ACE2 Activity Is Associated With an Increased Inflammatory Response in Smokers With COVID-19
Source: Front Physiol. 2021 May 28;12:653045. doi: 10.3389/fphys.2021.653045 (PMC8194708; doi:10.3389/fphys.2021.653045)
Supplement: Supplementary file 1 [file Table_1.docx]

**Supplementary Table 1:** Levels of various oxylipins in the patients sera from COVID-19 positive and COVID-19 recovered individuals

| **Analyte** | Mean(CoV Rec)+SD | Mean(CoV+)+SD |
| --- | --- | --- |
| **6-keto PGF_1α_** | 36.47+15.22 | 40.50+15.28 |
| **TXB_2_** | 13.41+15.40 | 19.00+15.56 |
| **PGF_2α_** | 0.53+0.34 | 1.12+0.67 |
| **PGE**_2_ | 3.75+1.50 | 10.71+12.16 |
| **12-HHTrE** | 44.28+51.21 | 44.29+27.56 |
| **LTB_4_** | 0.37+0.57 | 1.03+0.94 |
| **LXA_4_** | 0+0 | 0.78+1.73 |
| **5-HETE** | 0.91+1.46 | 4.15+5.27 |
| **11-HETE** | 7.61+5.53 | 22.15+25.92 |
| **12-HETE** | 444.69+224.49 | 725.52+418.16 |
| **15-HETE** | 19.42+9.51 | 41.55+23.57 |
| **20-HETE** | 0.31+0.15 | 0.49+0.52 |
| **5(6)-EET** | 5.93+6.10 | 13.33+8.10 |
| **8(9)-EET** | 27.47+28.80 | 54.11+36.75 |
| **11(12)-EET** | 75.28+61.32 | 180.54+191.52 |
| **9-HODE** | 19.82+28.23 | 28.98+50.18 |
| **13-HODE** | 46.19+63.19 | 46.87+58.15 |
| **17-HDHA** | 51.57+38.31 | 39.51+25.93 |

Concentrations are expressed as ng/mL
